# RETRACTED: Liu et al. The Effect of Intrinsic Mechanical Properties on Reducing the Friction-Induced Ripples of Hard-Filler-Modified HDPE. *Polymers* 2023, *15*, 268

**DOI:** 10.3390/polym15244708

**Published:** 2023-12-14

**Authors:** Chuanbo Liu, Chengqing Yuan, Shutian Liu

**Affiliations:** 1 School of Mechanical and Electronic Engineering, Wuhan University of Technology, Wuhan 430070, China; lchb72@whut.edu.cn; 2 School of Transportation and Logistics Engineering, Wuhan University of Technology, Wuhan 430063, China; ycq@whut.edu.cn; 3 Processing and Performance of Materials, Department of Mechanical Engineering, Eindhoven University of Technology, P.O. Box 513, 5600 MB Eindhoven, The Netherlands

The *Polymers* Editorial Office retracts the article “The Effect of Intrinsic Mechanical Properties on Reducing the Friction-Induced Ripples of Hard-Filler-Modified HDPE” [1] cited above.

Following publication, concerns relating to the accuracy and lack of appropriate permission to publish the data presented in this article [1] were raised to the editorial office.

Adhering to our complaints procedure, an investigation was conducted by the Editorial Office and Editorial Board. Following input from the Eindhoven University of Technology, it was confirmed that appropriate permission was not gained for a significant portion of the data presented and that the accuracy of certain elements of these data could not be verified. The article is therefore retracted.

This retraction was approved by the Editor in Chief of the journal *Polymers*.

The authors disagreed to this retraction.
